# RETRACTION: Incidence and Risk Factors of Delayed Wound Healing in Patients Who Underwent Unicompartmental Knee Arthroplasty

**DOI:** 10.1111/iwj.70605

**Published:** 2025-04-21

## Abstract

**RETRACTION:** LiJ. , JiaG. , DongW. , ZhaoF. , ZhaoZ. , YuX. , ZhuC. , LiJ. , LiuS. , JiangX. , and LiuG. , “Incidence and Risk Factors of Delayed Wound Healing in Patients Who Underwent Unicompartmental Knee Arthroplasty,” International Wound Journal 20, no. 2 (2023): 508–515, 10.1111/iwj.13898.35941751 PMC9885450

The above article, published online on 08 August 2022, in Wiley Online Library (http://onlinelibrary.wiley.com/), has been retracted by agreement between the journal Editor in Chief, Professor Keith Harding; and John Wiley & Sons Ltd. Following an investigation by the publisher, all parties have concluded that this article was accepted solely on the basis of a compromised peer review process. In addition, the investigation found that the authors provided incomplete informed consent information for the study. The editors have therefore decided to retract the article. The authors did not respond to our notice regarding the retraction.

**RETRACTION:** J. Li , G. Jia , W. Dong , F. Zhao , Z. Zhao , X. Yu , C. Zhu , J. Li , S. Liu , X. Jiang , and G. Liu , “Incidence and Risk Factors of Delayed Wound Healing in Patients Who Underwent Unicompartmental Knee Arthroplasty,” International Wound Journal 20, no. 2 (2023): 508–515, 10.1111/iwj.13898.35941751 PMC9885450

The above article, published online on 08 August 2022, in Wiley Online Library (http://onlinelibrary.wiley.com/), has been retracted by agreement between the journal Editor in Chief, Professor Keith Harding; and John Wiley & Sons Ltd. Following an investigation by the publisher, all parties have concluded that this article was accepted solely on the basis of a compromised peer review process. In addition, the investigation found that the authors provided incomplete informed consent information for the study. The editors have therefore decided to retract the article. The authors did not respond to our notice regarding the retraction.

